# Relationship Between FODMAP Diet and Irritable Bowel Syndrome: A Mendelian Randomization Study

**DOI:** 10.1002/fsn3.70037

**Published:** 2025-02-18

**Authors:** Lu Wang, Wei Cao, Qian‐Hua Zheng, Dehua Li, Yujun Hou, Shuai Chen, Fangli Luo, Xianjun Xiao, Ying Chen, Ying Li, Siyuan Zhou

**Affiliations:** ^1^ Hospital of Chengdu University of Traditional Chinese Medicine Chengdu Sichuan China; ^2^ Chengdu Integrated TCM and Western Medicine Hospital Chengdu China; ^3^ Chengdu University of Traditional Chinese Medicine Chengdu Sichuan Province China

**Keywords:** FODMAP diet, irritable bowel syndrome, Mendelian randomization

## Abstract

There is some evidence of a link between fermentable oligosaccharides, disaccharides, monosaccharides and polyols (FODMAP) diet and irritable bowel syndrome (IBS). However, few studies have analyzed the relationship between specific dietary intakes and IBS using Mendelian randomization (MR). Exposure and outcome datasets were sourced from the IEU Open GWAS project. Genetic variants significantly associated with 28 dietary intakes at a genome‐wide level were selected as instrumental variables. Summary statistics for the target outcome of IBS were obtained with a sample of 187,028 European individuals (4605 cases, 182,423 controls). Univariable and multivariable MR analyses were conducted to estimate the overall and independent MR associations after adjustment for genetic liability to intestinal flora. Genetic predispositions to six of 28 dietary intakes were associated with a decreased risk of IBS, including dried fruit, beef, cereal, lobster/crab, cereal, feta, and coffee, while cherry and poultry intake were associated with an increased risk of IBS. Three of eight associations persisted after adjusting for genetically predicted intestinal flora, and multivariable MR analysis identified that salad/raw vegetable intake was associated with a decreased risk of IBS. Twenty of 28 dietary intakes did not remain significantly associated after adjustment for intestinal flora. This study provides MR evidence supporting causal associations between FODMAP dietary intakes and IBS.

## Graphical abstract

Genetic predispositions to six of 28 dietary intakes were associated with a decreased risk of IBS, including dried fruit, beef, cereal, lobster/crab, cereal, feta, and coffee. Cherry and poultry intake were associated with an increased risk of IBS. Multivariable MR analysis identified that salad/raw vegetable intake was associated with a decreased risk of IBS after adjustment.

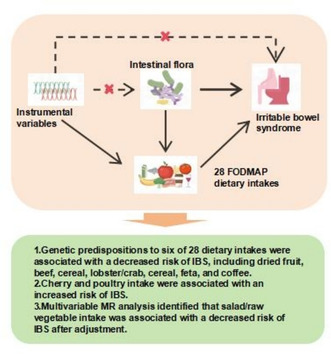

**Abbreviations**
CI: Confidence intervals
FFQ: Food frequency questionnaire
FODMAP: Fermentable oligosaccharides, disaccharides, monosaccharides and polyols
GFD: Gluten‐free diet
GWAS: Genome‐wide association study
IBS: Irritable bowel syndrome
IBS‐D: Diarrhea‐predominant IBS
IVs: Instrumental variables
IVW: Inverse variance weighted
LFD: Low FODMAP diet
MR: Mendelian randomization
MRC‐IEU: Medical Research Council Integrative Epidemiology Unit
MR‐PRESSO: Mendelian Randomization Pleiotropy RESidual Sum and Outlier
MVMR: Multivariable MR
OR: Odds ratios
SNPs: Single‐nucleotide polymorphisms
UKB: UK Biobank
UVMR: Univariable MR

## 1 Background

Irritable bowel syndrome (IBS) is a common functional gastrointestinal disorder characterized by chronic abdominal pain along with a change in bowel habit or frequent bloating (Enck et al. 2016). Recurrent symptoms significantly impair the quality of life of affected individuals, incurring a huge cost burden on the individual, the healthcare system, and society (Black and Ford 2020). The pathoetiology of IBS is complex and multifactorial and includes genetic factors, central nervous system function, diet, the microbiota, and alterations in intestinal permeability, immune function, and inflammatory pathways. Changes in gut microbiota composition are implicated as a main pathological regulator of IBS, and dietary habits that alter the gut microbiota composition have a significant impact on the disease course (Mars et al. 2020; Moayyedi et al. 2020). Indeed, dietary management remains the preferred treatment for most patients over a pharmacological approach, especially traditional dietary advice of a gluten‐free diet (GFD) or a low fermentable oligosaccharides, disaccharides, monosaccharides and polyols (FODMAP) diet (LFD) (Chey et al. 2022; Khalighi Sikaroudi et al. 2024; Sturkenboom et al. 2022). Current guidelines focus on LFD as the dietary treatment of choice for IBS, but one observational study showed that 84% of patients reported IBS symptoms triggered by eating certain foods, including some foods classified as LFDs (Böhn et al. 2013). Therefore, the exact relationships between specific foods and IBS must be established to refine dietary advice.

With limited evidence available from observational and interventional studies, Mendelian randomization (MR) is an epidemiological approach that provides the opportunity to examine potential causal effects between dietary factors and IBS (Davey Smith and Hemani 2014). Based on the concept of random assignment of alleles occurring naturally during meiosis, MR uses genetic variants associated with risk factors as instrumental variables (IVs), which minimizes the residual confounding in observational studies. A recent study showed that LFD alters the gut microbiota composition in patients with treated IBS while the post‐diet microbiota alters colonic gene expression in intestinal organ cultures (Bootz‐Maoz et al. 2022). Therefore, exploiting this well‐established genetic study design, we performed MR to examine the causal link between 28 FODMAP dietary intakes and risk of IBS (Davies et al. 2018; Pingault et al. 2018).

## 2 Methods

### 2.1 Study Design and Instrumental Variables Selection

**FIGURE 1:**
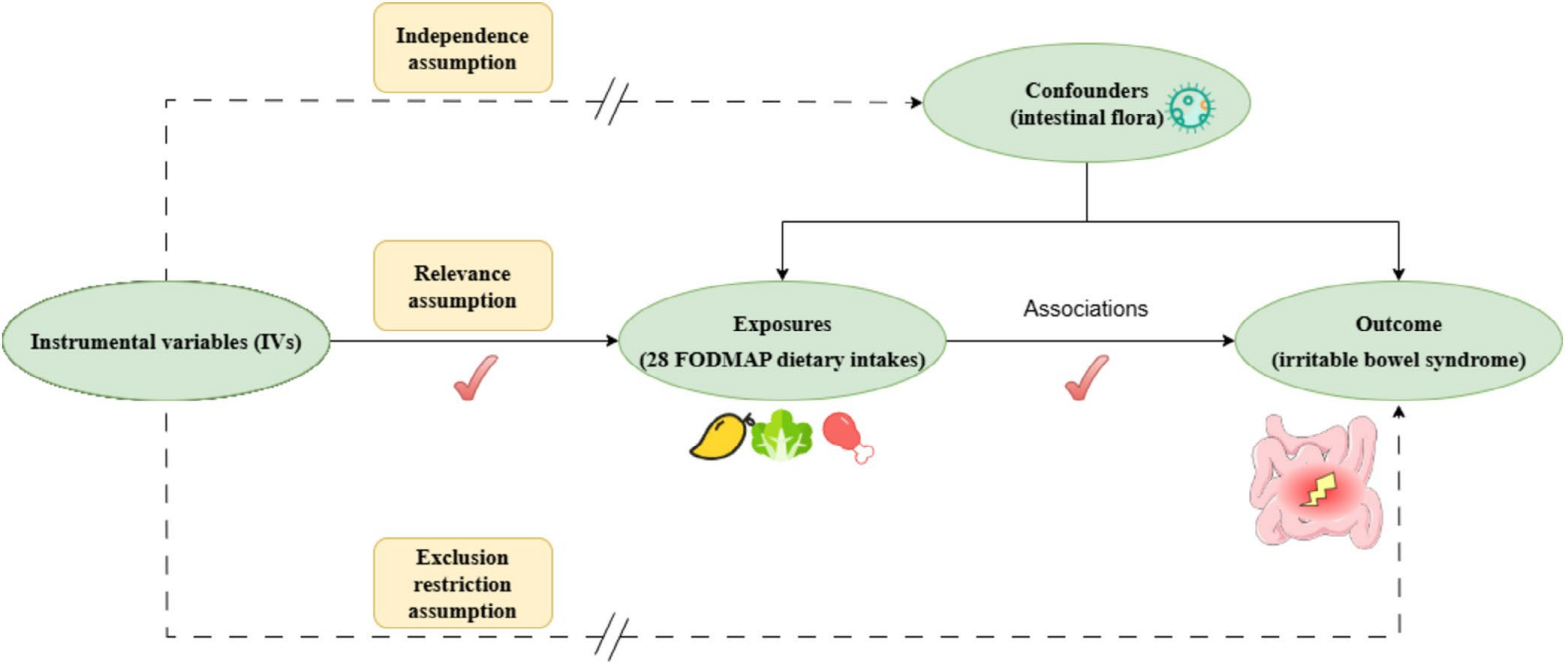
Study flow diagram and MR assumptions. (1) the relevance assumption: LVs should be strongly related to the corresponding phenotypes (28 dietary intakes); (2) the independence assumption: IVs must not be associated with any known or unknown confounders; (3) the exclusion restriction assumption: IVs affect the outcome (lBS) through the exposure rather than other pathways. FODMAP, fermentable oligosaccharides, disaccharides, monosaccharides and polyols.

This study was reported following the Strengthening the Reporting of Observational Studies in Epidemiology Using Mendelian Randomization guidelines (Skrivankova et al. 2021). Same as in conventional MR studies, we used the IVs as exposures, and potential mediators fulfilled three principal assumptions (independence, relevance, and exclusion restriction; Figure 1) (Burgess et al. 2015). We obtained genetic associations for 28 dietary intakes from large‐scale genome‐wide association study (GWAS) data generated by the Medical Research Council Integrative Epidemiology Unit (MRC‐IEU) in 2018. The exposure dataset included low FODMAP (bananas, broccoli, tomatoes, pork, beef, poultry, lamb, lobster/crab, fish, cheese, feta, bran cereal, tea, coffee, beer, and red wine), high FODMAP (apples, cherries, mangoes, garlic, celery, and milk), and others (fresh fruits, dried fruits, cooked vegetables, salads, and cereal). The data sources for exposures (i.e., 28 FODMAP dietary intakes) are provided in Table S1, and a list of corresponding food frequency questionnaire (FFQ) questions for each dietary intake are shown in Table S2 [see details in the UKB Data Showcase (http://biobank.ndph.ox.ac.uk/showcase/)].

According to the first MR assumption, we screened out significant autosomal biallelic single‐nucleotide polymorphisms (SNPs) exceeding the genome‐wide significance level (*p* < 5 × 10^−6^). We used a looser threshold to obtain more SNPs associated with the dietary phenotype, which has been used previously in many other MR studies (Lu et al. 2024; Su et al. 2024). Then, SNPs were pruned for independence using the clumping function in the “TwosampleMR” package (*r* ^2^ < 0.001 across a 10,000 kb distance) (Hemani et al. 2018). We also calculated the F‐statistic for each SNP's validity according to the formula: $F=\frac{N-1-k}{k}\times \frac{R^{2}}{1-R^{2}}$, where *N* is the sample size of the exposure, *k* is the number of IVs (*k* = 1 for each SNP), and *R* ^2^ is the variance explained by the SNPs calculated by $R^{2}=\frac{2\times {\text{Beta}}^{2}\times \;\left(1-\mathrm{EAF}\right)\times \mathrm{EAF}}{2\times {\text{Beta}}^{2}\times \left(1-\mathrm{EAF}\right)\times \mathrm{EAF}+2\times {\mathrm{SE}}^{2}\times \mathrm{EAF}\times \left(1-\mathrm{EAF}\right)\times N}$ (EAF, effect allele frequency; SE, standard error). SNPs with *F* < 10 were assessed as weakly IV‐biased and were removed in the next MR analysis (Burgess and Thompson 2011). IBS outcome data were from the latest summary statistics from FinnGen biobank analysis, which included 4605 IBS cases and 182,423 controls (https://gwas.mrcieu.ac.uk/datasets/finn‐b‐K11_IBS/). The diagnostic criteria for IBS were based on ICD‐10 standards. The GWAS was adjusted for sex, age, genotyping batch, the first 10 major components of the genetic data, and the genetic relatedness matrix.

### 2.2 Statistical Analysis

In the univariable MR (UVMR) analysis, we used the Wald ratio to calculate the effect estimates for the association between selected exposures and outcomes of each SNP. Four MR analytical methods were used to assess the causal effects of 28 FODMAP dietary intakes on IBS. Of these, the inverse variance weighted (IVW) method was mainly used to infer causality (Burgess et al. 2013). A *p*‐value < 0.05 was deemed significant. The results of causal associations are expressed as odds ratios (OR) and 95% confidence intervals (95% CI). The three other complementary calculations were the MR‐Egger method, weighted median method, and the weighted mode method (Bowden et al. 2015, 2016), where the same directions of results in both the complementary methods and the IVW estimate strengthened the validity of the effect estimates. After MR analysis, sensitivity analyses were performed, consisting of Cochran's *Q* test, the Mendelian Randomization Pleiotropy RESidual Sum and Outlier (MR‐PRESSO) global test, and the MR‐Egger intercept test. In Cochran's *Q* test, the heterogeneity statistic *Q* was calculated for the effect estimates by IVW and MR‐Egger methods. A large *Q* statistic value and a *p*‐value < 0.05 suggested the existence of bias and heterogeneity. Then, the MR‐PRESSO global test and MR‐Egger intercept test were used to detect horizontal pleiotropy (Verbanck et al. 2018). *p*‐values > 0.05 indicated the absence of horizontal pleiotropy across the analyses.

In the multivariable MR (MVMR) analysis, considering that the intestinal flora is strongly associated with dietary intake, which in turn is associated with IBS, we performed a regression‐based multivariate MR analysis, adjusting for the three reported bacterial traits (Liu et al. 2023). The genetic association of these IVs with the three bacterial traits was also derived from a publicly available GWAS dataset (GWAS ID: phylum Actinobacteria: ebi‐a‐GCST90017110; genus *Eisenbergiella*: ebi‐a‐GCST90016991; genus *Flavonifractor*: ebi‐a‐GCST90017010). We consolidated all genetic instruments, eliminated duplicate SNPs, and excluded correlated SNPs (*r* ^2^ < 0.001), retaining those with the *p*‐value (*p* < 5 × 10^−6^) for genetic association with each trait. Associations of the remaining SNPs with the exposures and the outcome were then extracted and used to fit multivariable models. The IVW method was used to overcome potential confounding and to independently estimate the causal effect of multiple exposure variables on the outcome.

All statistical analyses and visualizations were performed in R software (v4.2.2 for Windows) with the main packages “TwoSampleMR,” “data.table,” “R.utils,” and “dplyr.”

## 3 Results

We obtained 4 to 199 SNPs associated with each of 28 dietary intakes as IVs meeting universally accepted genome‐wide significance thresholds (*p* < 5 × 10^−6^, *r* ^2^ < 0.001, kb = 10,000). Each IV was valid with an F‐statistic > 10, indicating a low risk of bias from weak instruments (Tables S3–S5).

**FIGURE 2:**
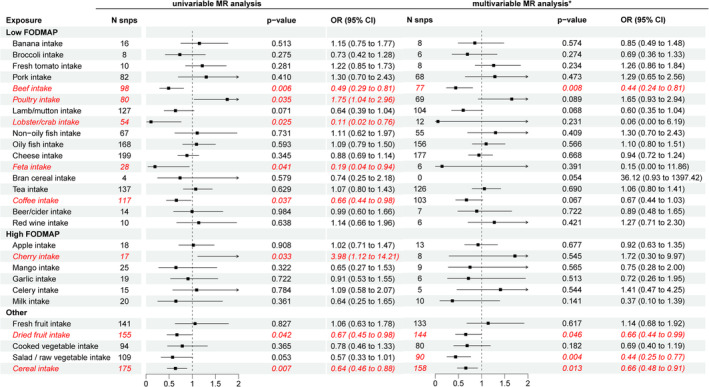
Mendelian randomization associations of FODMAP Diet with IBS. CI, confidence interval; MR, Mendelian randomization; OR, odds ratio; SNP, single nucleotide polymorphism. FODMAP, fermentable oligosaccharides, disaccharides, monosaccharides and polyols. *Multivariable MR analysis adjusted for intestinal flora. Red markings indicate dietary intakes associated with IBS risk.

Figure 2 shows the associations between 8 of the 28 genetically predicted dietary intakes and IBS in the UVMR analysis. Genetic predisposition to beef (OR, 0.49; 95% CI, 0.29 to 0.81; *p* = 0.006), lobster/crab (OR, 0.11; 95% CI, 0.02 to 0.76; *p* = 0.025), feta (OR, 0.19; 95% CI, 0.04 to 0.94; *p* = 0.041), coffee intake (OR, 0.66; 95% CI, 0.44 to 0.98; *p* = 0.037), dried fruit (OR, 0.67; 95% CI, 0.45 to 0.98; *p* = 0.041), and cereal (OR, 0.64; 95% CI, 0.46 to 0.88; *p* = 0.007) were associated with a lower odds of IBS while genetic predisposition to poultry intake (OR, 1.75; 95% CI, 1.04 to 2.96; *p* = 0.035) and cherry (OR, 3.98; 95% CI, 1.12 to 14.21; *p* = 0.033) was associated with a higher odds of IBS. There was little evidence to support genetically predicted associations of the other 20 dietary intakes (fresh fruit, apple, banana, mango, cooked vegetables, salad/raw vegetables, broccoli, celery, garlic, fresh tomato, pork, lamb/mutton, non‐oily fish, oily fish, plain cereal, bran cereal, milk, cheese, tea, beer/cider, and red wine) and the risk of IBS.

The results from weighted median and weighted mode analyses were generally consistent with IVW. The MR‐Egger method showed similar effects of some dietary intakes on IBS; however, some of these associations were weakened due to the wider CIs (Table S6). The MR‐Egger intercept tests indicated horizontal pleiotropy for coffee intake (*p*‐value for MR‐Egger intercept < 0.05) but not for other outcomes. All *p*‐values were > 0.05 in the MR‐PRESSO global test and Cochran's *Q* test, suggesting that there was no significant horizontal pleiotropy (Table 1).

**TABLE 1:** Sensitivity analyses of associations between FODMAP Diet and IBS.

Outcomes	Methods	Cochran's *Q* test		MR‐Egger		MR‐PRESSO
		*Q*	*p*	Intercept	*p*	*p* of global test
*Low FODMAP*						
Banana intake	MR Egger	18.526	0.184	−0.013	0.579	0.227
	IVW	18.953	0.216			
Broccoli intake	MR Egger	1.992	0.920	−0.001	0.966	0.971
	IVW	1.994	0.960			
Broccoli intake	MR Egger	1.992	0.920	−0.001	0.966	0.971
	IVW	1.994	0.960			
Pork intake	MR Egger	83.270	0.379	0.006	0.532	0.418
	IVW	83.680	0.397			
Beef intake	MR Egger	100.883	0.347	0.013	0.137	0.293
	IVW	103.252	0.313			
Poultry intake	MR Egger	81.423	0.373	0.001	0.891	0.414
	IVW	81.443	0.403			
Lamb/mutton intake	MR Egger	123.939	0.510	−0.006	0.439	0.522
	IVW	124.541	0.520			
Lobster/crab intake	MR Egger	68.332	0.064	−0.003	0.880	0.079
	IVW	68.362	0.076			
Non‐oily fish intake	MR Egger	59.515	0.669	0.007	0.422	0.66
	IVW	60.167	0.679			
Oily fish intake	MR Egger	173.073	0.338	0.016	0.042	0.276
	IVW	177.435	0.276			
Cheese intake	MR Egger	205.698	0.321	−0.002	0.799	0.345
	IVW	205.766	0.338			
Feta intake	MR Egger	28.437	0.337	−0.017	0.351	0.319
	IVW	29.424	0.341			
Bran cereal intake	MR Egger	3.762	0.152	−0.032	0.823	0.478
	IVW	3.883	0.274			
Tea intake	MR Egger	124.208	0.737	−0.007	0.235	0.728
	IVW	125.634	0.727			
Coffee intake	MR Egger	113.369	0.526	−0.014	0.009	0.348
	IVW	120.401	0.371			
Beer/cider intake	MR Egger	20.707	0.055	−0.020	0.548	0.066
	IVW	21.366	0.066			
Red wine intake	MR Egger	9.496	0.302	−0.035	0.369	0.357
	IVW	10.572	0.306			
*High FODMAP*						
Apple intake	MR Egger	9.683	0.883	0.003	0.908	0.92
	IVW	9.697	0.916			
Cherry intake	MR Egger	15.128	0.442	0.006	0.739	0.514
	IVW	15.244	0.507			
Mango intake	MR Egger	24.231	0.391	0.000	0.984	0.528
	IVW	24.231	0.448			
Garlic intake	MR Egger	15.853	0.534	0.031	0.250	0.531
	IVW	17.270	0.505			
Celery intake	MR Egger	17.535	0.176	0.030	0.157	0.109
	IVW	20.577	0.113			
Milk intake	MR Egger	26.730	0.084	−0.021	0.297	0.075
	IVW	28.444	0.075			
*Other*						
Fresh fruit intake	MR Egger	133.460	0.617	0.000	0.967	0.654
	IVW	133.461	0.639			
Dried fruit intake	MR Egger	163.461	0.267	0.000	0.969	0.304
	IVW	163.462	0.286			
Cooked vegetable intake	MR Egger	75.564	0.893	−0.003	0.681	0.886
	IVW	75.735	0.904			
Salad/raw vegetable intake	MR Egger	93.630	0.818	0.000	0.954	0.861
	IVW	93.633	0.836			
Cereal intake	MR Egger	145.321	0.938	0.007	0.287	0.933
	IVW	146.461	0.936			

Abbreviations: FODMAP, fermentable oligosaccharides, disaccharides, monosaccharides and polyols; IVW, inverse variance‐weighted; MR, Mendelian randomization; MR‐PRESSO, Mendelian Randomization Pleiotropy RESidual Sum and Outlier.

In the MVMR analysis adjusted for three genetically predicted bacterial traits (phylum Actinobacteria, genus *Eisenbergiella*, and genus *Flavonifractor*), the associations between genetically predicted beef (OR, 0.44; 95% CI, 0.24 to 0.81; *p* = 0.008), dried fruit (OR, 0.66; 95% CI, 0.44 to 0.99; *p* = 0.046) or cereal intake (OR, 0.66; 95% CI, 0.48 to 0.91; *p* = 0.013) and IBS were consistent with results from the UVMR analysis. However, the other five dietary intakes associated with IBS risk in UVMR analysis were not genetically linked after adjustment (Table S7). Interestingly, MVMR analysis found that the genetic susceptibility to salad/raw vegetable intake was negatively associated with risk of IBS (OR, 0.44; 95% CI, 0.25 to 0.77; *p* = 0.004) (Figure 2).

## 4 Discussion

To our knowledge, this is the first study to explore the causal relationships between 28 specific FODMAP dietary intakes and IBS using MR methods in a large sample, and the summary results of this analysis are shown in Figure 2. We found robust associations between genetic predisposition to intake of dried fruit, beef, or cereal and decreased risk of IBS, both in UVMR analysis and MVMR analysis. In addition, the univariable MR analysis found a causal relationship between intake of poultry, lobster/crab, feta, coffee, or cherry and risk of IBS. MVMR analysis also identified an association between salad/raw vegetable intake and IBS after adjustment for the effect of intestinal flora. Moreover, intake of fresh fruit, apple, banana, mango, cooked vegetables, broccoli, celery, garlic, fresh tomato, pork, lamb/mutton, non‐oily fish, oily fish, plain cereal, bran cereal, milk, cheese, tea, beer/cider, and red wine was not associated with IBS.

An increased appreciation of how dietary intake affects pathophysiology plays an increasingly important role in our understanding and treatment of IBS. Recent studies have shown that the LFD diet significantly improves the global symptoms and bowel habits of patients with IBS, particularly diarrhea‐predominant IBS (IBS‐D) (Guerreiro et al. 2020; van Lanen et al. 2021; Wang et al. 2021). The acronym FODMAP denotes all foods containing fermentable oligosaccharides, disaccharides, monosaccharides, and polyphenols, and common FODMAPs include fruits, vegetables, dairy products, and grains. Regarding the underlying mechanism, reducing the intake of fermentable carbohydrates, fiber, and fructans reduces the amount of substrate available for colonic fermentation and reduces intestinal osmolality and gas production, thus helping to reduce gastrointestinal symptoms (Barrett et al. 2010; Bootz‐Maoz et al. 2022; Staudacher et al. 2012). In addition, a high fructose diet can cause microbial dysbiosis progressing to barrier deterioration, low‐grade intestinal inflammation, and endotoxemia (Todoric et al. 2020). Therefore, among the available options, elimination of fermentable FODMAPs from the diet is currently the most evidence‐based dietary treatment for patients with IBS. Similarly, we observed that the intake of beef, lobster/crab, cereal, feta, and coffee with low FODMAP content was associated with reduced IBS risk, whereas the intake of cherries with their high FODMAP content was associated with increased IBS risk. For coffee, a cross‐sectional study in Iran found that individuals who drank coffee weekly or more frequently had a greater risk of IBS than those who never drank coffee (OR, 1.44; 95% CI: 1.02 to 2.04) (Koochakpoor et al. 2021). There are several reasons for these inconsistent results. First, observational studies have been based on West Asian populations, and the results may not apply to other ethnic groups. Indeed, a study based on an ongoing large prospective cohort, the UK Biobank (UKB) study, found that coffee intake reduces IBS risk in a European population. Second, we did not analyze the specific effect of coffee or caffeine intake levels (Wu et al. 2023). Third, retrospective studies with their small sample sizes may have unmeasured confounding factors. Interestingly, we also found that dietary intake of poultry, often categorized as low FODMAP, was associated with a high risk of IBS. No direct evidence exists to support an association between the poultry diet and IBS. Some studies have reported that some foodborne pathogens with poultry as host are closely related to IBS, such as Campylobacteriosis (Facciolà et al. 2017; Wagenaar et al. 2013). In addition, poultry intake may be representative of other dietary factors or lifestyle habits independently associated with IBS. For example, individuals who consume more poultry may also consume more of other dietary elements, such as processed foods, which may increase the risk of IBS (Chen et al. 2020). Also, poultry preparation methods (e.g., frying or seasoning) may introduce FODMAPs or other irritants (Rijnaarts et al. 2021).

Additionally, the intake of broccoli, tomato, pork, lamb/mutton, fish, cheese, tea, beer/cider, and red wine with low FODMAP content and apple, banana, mango, celery, garlic, bran cereal, and milk with high FODMAP content was not associated with IBS risk in our study. We hypothesized that the effects of these foods on IBS may vary depending on the amount consumed, the effects of other components of the food (e.g., dietary antioxidants, dietary acid load), processing methods, or individual differences in gut flora (Mobasheri et al. 2022; Saneie et al. 2022). Low FODMAP foods may not significantly affect the fermentation products of the gut or the composition of the intestinal flora, resulting in a weaker trigger for IBS (Jacqueline Susanne Barrett and Gibson 2007). Similarly, the intake of a single high FODMAP food may not be sufficient to significantly affect osmolality or gas production in the intestinal lumen (Gibson and Shepherd 2010). There is also the possibility that existing sample sizes and genetic tools may not be sufficient to detect the subtle effects of some of these foods. Further, long‐term perspective studies are needed to validate the findings.

It is well known that there is a two‐way interaction between diet and the intestinal flora, and they are key regulators in the pathogenesis of IBS. Dietary components can directly alter the composition and metabolic function of the gut microbiota. For example, high‐fat diets may exacerbate IBS symptoms and inflammation levels by altering bile acid metabolism and enriching pro‐inflammatory flora, such as Firmicutes and Proteobacteria (O'Mahony et al. 2023). Conversely, a diet rich in anti‐inflammatory components, such as polyphenols and omega‐3 fatty acids, can help reduce IBS symptoms by promoting the growth of anti‐inflammatory flora, such as short‐chain fatty acid‐producing bacteria (Garcia‐Mantrana et al. 2018). In addition, dietary components rely on secondary metabolites (e.g., short‐chain fatty acids, bile acids, and polyphenol metabolites) produced by the intestinal flora, which in turn affects the intestinal environment and IBS symptoms (Bolte et al. 2021; Garcia‐Mantrana et al. 2018). The independence assumption of MR analysis stipulates that IVs cannot be associated with other confounding factors. Therefore, we performed MVMR analysis by adjusting for intestinal flora and found a robust causal relationship between dried fruit, salad/raw vegetable, beef, and cereal intake and IBS risk. To our knowledge, no causal relationship between dried fruits and IBS has previously been reported. Dried fresh fruits retain micronutrients and bioactive compounds such as minerals, vitamins, flavonoids, and carotenoids (Sadler et al. 2019), and we hypothesize that the protective effect of dried fruit intake on IBS may be related to the anti‐inflammatory and antioxidant effects of the micronutrients and bioactive compounds present in dried fruits (Di Lorenzo et al. 2016; Donno et al. 2019; Puglisi et al. 2008). Salad/raw vegetable intake is commonly believed to exacerbate diarrhea and abdominal pain symptoms in patients with IBS, but we found it to be a protective factor. On the one hand, it has been suggested that lettuce‐based salads are not the main cause of IBS risk but rather the addition of ingredients such as onions, peppers, and salad dressings, although lettuce with tomatoes, hard‐boiled egg slices, and oil and vinegar as the dressing have been shown to alleviate IBS (MacDermott 2007). On the other hand, there is also evidence that higher phytochemical intake is associated with reduced IBS severity, especially in women (Lari et al. 2023). With respect to beef, a case–control study found that lower levels of IgG antibodies targeting beef were significantly associated with the severity of IBS symptoms (OR, 0.75; 95% CI, 0.60 to 0.94; *p* = 0.012) (Ligaarden et al. 2012). Unlike observational studies, our MR analysis found that beef intake may reduce IBS risk, the differences possibly due to the more restricted study area and small sample size, which may have confounding factors and specificity. Furthermore, not all patients with IBS have food allergies, and the IgG test is not specific for the diagnosis of IBS. With respect to cereal intake, the consumption of fructan‐containing cereal products does not benefit IBS patients. As a result, cereal‐based products with low FODMAPs have been developed (Galgano et al. 2023; Schmidt and Raczyk 2023); for example, yeast convertase‐mediated hydrolysis of fructan has been controlled during dough fermentation, thereby regulating fructose concentrations (Laurent et al. 2020). Tritordeum (a Spanish cereal)‐based foods have also been shown to support epithelial barrier function and integrity, greatly reducing symptoms and improving the psychosocial profile of IBS‐D patients (Riezzo et al. 2023; Russo et al. 2022).

The main strength of this study is the MR design, which minimizes bias from confounding and reverse causality, thus improving the causal inference of the association between certain food intakes and IBS. We also performed MVMR analysis to avoid false‐positive inferences and further adjusted for potential confounders to ensure careful interpretation of the results. In addition, we restricted the analysis to individuals of European ancestry, which minimized population stratification bias.

This study has several limitations. First, we selected the IVs for food intakes at a *p*‐value threshold of < 5.0 × 10^−6^, which is larger than the traditional genome‐wide significance level (*p* < 5 × 10^−8^), to obtain sufficient IVs. Second, we could not perform analyses for different IBS subtypes because this information was not available. Third, this study only included European populations, which limits the applicability of our findings to other populations. Further research is needed in the future in ethnically and geographically diverse populations to increase the extrapolation of the results. Fourth, in addition to intestinal flora, other possible influences such as the quantity consumed, other ingredients in the food, and processing methods were not analyzed.

## 5 Conclusions

In conclusion, our MR investigation provided evidence that genetic susceptibility to seven food intakes (beef, lobster/crab, feta coffee, dried fruit, salad, and cereal) were associated with decreased risks of IBS, and two genetically predicted food intakes (poultry and cherry) were associated with increased risks of IBS. Intestinal flora appeared to mediate many of these associations.

## Author Contributions

**Lu Wang:** conceptualization (equal), writing – original draft (equal). **Wei Cao:** conceptualization (equal), writing – original draft (equal). **Qian‐Hua Zheng:** data curation (equal), software (equal). **Dehua Li:** formal analysis (equal), writing – original draft (equal). **Yujun Hou:** data curation (equal). **Shuai Chen:** resources (equal), software (equal). **Fangli Luo:** validation (equal). **Xianjun Xiao:** visualization (equal). **Ying Chen:** data curation (equal). **Ying Li:** investigation (equal), project administration (equal), writing – review and editing (equal). **Siyuan Zhou:** conceptualization (equal), resources (equal), writing – review and editing (equal).

## Ethics Statement

The authors have nothing to report.

## Conflicts of Interest

The authors declare no conflicts of interest.

## Supporting information


**Table S1.** GWAS data sources used in the study.
**Table S2.** food frequency questionnaire questions for each dietary intake.
**Tables S3–S5.** The characteristics of the instrumental variables (IVs).
**Table S6.** Univariable Mendelian randomization analysis results for IVs.
**Table S7.** Multivariable Mendelian randomization analysis results for IVs.

## Data Availability

Data described in the manuscript, code book, and analytic code will be made available upon request pending application and approval.
